# Peer review of "The Decay of Pertussis Antibodies in Children Aged 0–14 Years in Jiangsu Province, China"

_vaccines, 2023, doi:10.3390/vaccines11081336_

Round 1

Reviewer 1 Report

The introduction is very well-written and quite exhaustive and informative.  It points out, however that many of the results of the current study are not very different than what is already in the literature; antibody levels wane with time after immunization and this likely is a culprit in the resurgence of pertussis.

Although somewhat obvious what they are, the Y axes in Figure 1 should be labelled.

Please define “IU” in IU/ml.  Many readers will know the meaning, some will not.

Line 137.  Varicella specific IgG antibodies?  Why?  Is this an error?

Iine 178.  Is the epidemic 2018 as the text states or 2019 as Figure 1 indicates?

The use of 3 shades of blue in these graphs makes it a bit difficult to discern whether the peak in age at about 5 years of age is from 2007-2012 or 2022.  Colors can be kept if desired, but please put symbols within the lines to make it easier to interpret as a stand-alone figure.

Table 1 is difficult to interpret as  “Positivity rate (%)……..Infection Rate (%)” has number (number) below.  e.g.,  in 0-2m age group is the <5 IU/ml value  of “13(68.4)” meaning 13 of 19 children had anti-PT antibodies at that level and 68.4 of these children were infected?  I don’t think this is what the authors are stating, but it is difficult to discern what they  mean. Why is there an Infection Rate column associated with the anti-PT antibodies, but not with the anti FHA antibodies section of the table?   Great info in this table I think, but it needs much better formatting.

Figure 3 legend needs a much more clear explanation.  It is unclear what the authors are stating about this graph.  The text does a somewhat better job at explaining the graphs, but these graphs will be informative to only a small subset of readers without a more clear explanation.

The discussion is well-written, but too long.  It needs to be reduced at least by 30%. 

There is no mention of adult cases of pertussis in the region and the role of adult infections in the resurgence of this disease

with only a couple of spots where edits may be appropriate, this was an exceptionally well-written paper in terms of English usage

Author Response

  1. Although somewhat obvious what they are, the Y axes in Figure 1 should be labelled.

 Reply: The modified figure 1 was as follows:

  1. Please define “IU” in IU/ml.  Many readers will know the meaning, some will not.

 Reply: We have added an explanation of IU in the laboratory assays section of the manuscript.

IU stands for "International Unit." In the medical and biological fields, IU is a standardized unit of measurement for biological activity. Due to slight variations in different manufacturers' test kits, using different units to express results can lead to confusion. Therefore, the use of international units helps standardize measurement results, allowing for comparisons and sharing of results between different laboratories. For the antibody testing of a vaccine's antibody levels using a test kit, IU/mL represents the quantity of antibodies in one milliliter (mL) of blood sample, standardized in international units. Typically, higher IU/mL values indicate a higher concentration of specific antibodies in a person's body, indicating a stronger immune response to the vaccine or disease. Different vaccines or antibody tests may have different IU/mL reference ranges, and the specific reference range should be provided by the manufacturer or healthcare professionals when using the test kit.

  1. Line 137.  Varicella specific IgG antibodies?  Why?  Is this an error?

 Reply: I am very sorry that I failed to find out and correct this obvious error when uploading the manuscript, it should be pertussis specific IgG antibodies.

  1. Iine 178.  Is the epidemic 2018 as the text states or 2019 as Figure 1 indicates?

 Reply: Modify as follows:

During the study period, we collected data on 1959 confirmed cases of pertussis among children aged 0-14 years. Our analysis revealed two distinct pertussis epidemics, one in 2019 and another in 2022 (Figure 1).

  1. The use of 3 shades of blue in these graphs makes it a bit difficult to discern whether the peak in age at about 5 years of age is from 2007-2012 or 2022.  Colors can be kept if desired, but please put symbols within the lines to make it easier to interpret as a stand-alone figure.

 Reply: The modified figure 2 was as follows:

  1. Table 1 is difficult to interpret as  “Positivity rate (%)……..Infection Rate (%)” has number (number) below.  e.g.,  in 0-2m age group is the <5 IU/ml value  of “13(68.4)” meaning 13 of 19 children had anti-PT antibodies at that level and 68.4 of these children were infected?  I don’t think this is what the authors are stating, but it is difficult to discern what they  mean. Why is there an Infection Rate column associated with the anti-PT antibodies, but not with the anti FHA antibodies section of the table?   Great info in this table I think, but it needs much better formatting.

Reply: According to the manufacturer's instructions, the cut-off value for positive anti-PT and anti-FHA IgG serum was 20 IU/ml (IU/mL represents the quantity of antibodies in one milliliter of blood sample, standardized in international units), and ≤20 IU/ml was considered negative. The minimum level of anti-PT IgG detection was 5 IU/ml(please see Laboratory Assay section). The <5 IU/ml value of “13(68.4)” meaning 13 of 19 children were undetectable in 0-2m age group. According to the manufacturer's instructions, an anti-PT IgG concentration ≥80 IU/mL was considered to indicate a recent infection if the subject had not received the pertussis containing vaccine within the previous year. Anti FHA antibodies weres not the most specific indicator of whooping cough, so there was no specific cut-off value for anti FHA antibody that suggests recent infection.

  1. Figure 3 legend needs a much more clear explanation.  It is unclear what the authors are stating about this graph.  The text does a somewhat better job at explaining the graphs, but these graphs will be informative to only a small subset of readers without a more clear explanation.

 Reply: the legend of Figure 3 is modified as follows: The duration after immunization was determined according to the minimum OR (OR = 1) of RCS model fitting, the OR can reflect the relationship between duration after immunization and IgG antibody concentrations (≥20 IU/mL). A graph was then plotted with post-immunization duration on the x-axis and the OR value for the model-predicted PT -IgG antibody concentration ≥ 20IU/mL on the y-axis. The color intervals represent the 95% confidence interval for the OR. (A) The duration after immunization and the OR of having PT-IgG antibody concentrations ≥20 IU/mL in children with documented immunization history (1-4 doses). (B) The duration after immunization and the OR of having FHA-IgG antibody concentrations ≥20 IU/mL in children with documented immunization history (1-4 doses). (C) The duration after immunization and the OR of having PT-IgG antibody concentrations ≥20 IU/mL in children with a full course of pertussis vaccination (4 doses). (D) The duration after immunization and the OR of having FHA-IgG antibody concentrations ≥20 IU/mL in children with a full course of pertussis vaccination (4 doses).

  1. The discussion is well-written, but too long.  It needs to be reduced at least by 30%. 

 Reply: To make the Discussion section of the manuscript more concise and accessible, we shortened the manuscript from 1437 words to 1096.please see page 16-19.

  1. There is no mention of adult cases of pertussis in the region and the role of adult infections in the resurgence of this disease

 Reply: In this study, we focused on the decline of pertussis antibody levels in the 0-14 age group. This is because children are the primary population affected by pertussis infection and have significant implications for vaccine administration and disease transmission. While the pertussis antibody levels and infection status in adults are also important research topics, they were not included in this study due to limitations in scope and length. Furthermore, adult pertussis infections play a crucial role in the resurgence of pertussis cases. The immune status and pertussis antibody levels in adults can also impact the immunity protection of children. Hence, future research could further investigate the precise role of adult pertussis antibody levels and infections in pertussis case resurgence, and consider them in the formulation of pertussis prevention and control strategies. We will consider this section as a limitation of this manuscript. please see page 18.

Reviewer 2 Report

In this manuscript, the authors investigated the factors influencing pertussis toxin (PT) and filamentous hemagglutinin (FHA) antibody levels in children aged 0-14 years in Jiangsu Province. They also analyzed the changes in IgG antibody levels after pertussis vaccination using a restricted cubic spline (RCS)-fitted binary logistic regression model. The findings indicated that children with a history of immunization experienced a significant drop in antibody levels around 17 months after the last dose of the vaccine. Therefore, the authors recommend administering pertussis-containing vaccines as booster immunizations for older children. This study is important for monitoring pertussis vaccination response and has implications for vaccination strategy design. However, the data used to support the conclusion should be carefully evaluated.

Here are some major points to consider:

1. The title of the manuscript does not accurately reflect the main finding. The study focuses on four cities in Jiangsu Province, not the entire east of China.

2. It is unclear why the mention of the COVID-19 pandemic is included in the article. The study investigates the changes in epidemiological characteristics of pertussis in Jiangsu Province from 2007 to 2022, and the results are unrelated to the COVID-19 pandemic.

3. The rationale for detecting pertussis toxin (PT) and filamentous hemagglutinin (FHA) antibodies should be provided. Are these antigens included in the vaccines or are they main antigens during infection? This information is crucial for understanding the results.

4. Some background information is needed to explain the relationship between PT antibody, FHA antibody, and vaccine protection. Are these antibodies indicative of vaccine protection or do they simply represent antigenicity and human response to the vaccines?

5. In line 136, why varicella-specific IgG antibodies were detected?

6. In line 142, “The serum was diluted from 50 to 6400-fold, 100 to 12800-fold, and 100 to 12800-fold for testing PT and FHA,” is confusing. The authors should provide more clarity or revise the statement.

7. The authors should explain in detail how the exact concentration of serum IgG was calculated and provide guidelines for interpreting the results. For example, it is mentioned that <5 IU/ml was considered negative, but what about concentrations between 5-20 IU/ml and >20 IU/ml? Additionally, the rationale for considering ≥80 IU/ml as indicating a recent infection should be provided.

8. Line 155: It is not clear how the geometric mean concentrations (GMCs) and corresponding seropositivity rates of antibodies were calculated. The authors should explain the methodology in more detail.

9. Figure 2: The authors should consider improving the display of the results to make them clearer and easier to understand.

10. P8 line 14: The statement about the proportion of individuals with anti-PT IgG antibody levels in different age groups seem not consistent with the results in Table 1. Please carefully review the data.

11. P8 line 17: The authors should specify which group the result of the GMC of anti-FHA IgG antibody and the seropositivity rate corresponds to.

12. Table 1: The definition of <5IU/ml as negative should be reflected in the appropriate column heading.

13. P8 line 22: “Specifically, the seropositivity rate in the group receiving the full course vaccination was 43.3%, while in other groups it was 24.6% (Table 1)”. 24.6% was for which groups in Table 1? The authors should review the data and revise the statement about the seropositivity rate in different groups to ensure consistency with Table 1. Additionally, they should clearly define how the seropositivity rate was calculated.

14. P8 line 31: The authors should specify what 17.71 months and 16.95 months refer to which group.

15. P8 line 37: Similarly, the authors should provide clarification about the time intervals mentioned (e.g., 7.36 months and 7.56 months) to which group.

16. Figure 3: The authors should clearly indicate the unit for the x-axis, which represents the time since the last dose of the vaccine. It would also be helpful to label the subpanels of the figures with keywords to make them easily understandable.

17.The overall populated tested include the kids with full shot and some with uncompleted shot. The relative low positivity rate of antibody is due to the uncompleted shot or the decline of the antibody? It would be valuable for the authors to conduct further analysis within the group of fully vaccinated children to assess the antibody trends with age. This would help elucidate whether the relative low positivity rate of antibodies is due to incomplete vaccination or a decline in antibody levels.

Author Response

  1. The title of the manuscript does not accurately reflect the main finding. The study focuses on four cities in Jiangsu Province, not the entire east of China.

 Reply: We changed the title of the manuscript to ‘the Decay of Pertussis Antibodies in 0-14 Age in Jiangsu of China during the COVID-19 Pandemic’, please see page 1.

  1. It is unclear why the mention of the COVID-19 pandemic is included in the article. The study investigates the changes in epidemiological characteristics of pertussis in Jiangsu Province from 2007 to 2022, and the results are unrelated to the COVID-19 pandemic.

 Reply: Thank you for your valuable suggestions. This study investigates the epidemiological changes in pertussis in Jiangsu Province from 2007 to 2022. It aims to elucidate the prevalence of pertussis in the region and provide readers with an understanding of the current trends in pertussis occurrence, including the period during the COVID-19 pandemic. The primary focus of this research lies in evaluating pertussis antibody levels in the 0-14 age group by analyzing blood specimens collected from healthy individuals (aged 0-14 years) during the period of 2019-2022 (the COVID-19 pandemic). The data were analyzed using a binary logistic regression model with restricted cubic splines (RCS) to study the dynamic changes in IgG antibody levels after pertussis vaccination. Notably, this study did not compare the impact of the COVID-19 pandemic on pertussis prevalence. Accordingly, the revised title of this manuscript is " The Decay of Pertussis Antibodies in 0-14 Age in Jiangsu, China " please see page 1.

  1. The rationale for detecting pertussis toxin (PT) and filamentous hemagglutinin (FHA) antibodies should be provided. Are these antigens included in the vaccines or are they main antigens during infection? This information is crucial for understanding the results.

 Reply: The preparation of DTaP adopts co-purification technology, in which Pertussis toxin (PT) and filamentous hemagglutinin (FHA) are the main components, which are responsible for generating the immune response, please see page 2.

  1. Some background information is needed to explain the relationship between PT antibody, FHA antibody, and vaccine protection. Are these antibodies indicative of vaccine protection or do they simply represent antigenicity and human response to the vaccines?

Reply: The serum titers of pertussis IgG antibodies against PT and FHA are assessed to monitor vaccine-induced herd immunity. At the same time, we also cite the corresponding literature to support this statement.

[1] Konda T, Kamachi K, Iwaki M, Matsunaga Y. Distribution of pertussis antibodies among different age groups in Japan. Vaccine 2002;20:1711–7.

[2] Xu Y, Wang L, Xu J, Wang X, Wei C, Luo P, et al. Seroprevalence of pertussis in China: need to improve vaccination strategies. Hum Vaccin Immunother 2014;10:192–8.

[3] Van der Wielen M, Van Damme P, Van Herck K, Schlegel-Haueter S, Siegrist CA. Seroprevalence of Bordetella pertussis antibodies in Flanders (Belgium). Vaccine 2003;21:2412–7.

  1. In line 136, why varicella-specific IgG antibodies were detected?

Reply: I am very sorry that I failed to find out and correct this obvious error when uploading the manuscript, it should be pertussis specific IgG antibodies.

6.In line 142, “The serum was diluted from 50 to 6400-fold, 100 to 12800-fold, and 100 to 12800-fold for testing PT and FHA,” is confusing. The authors should provide more clarity or revise the statement.

Reply: The sera were diluted by 1:100 and tested in duplicate for presence of total anti-PT and FHA IgG antibodies. Photometric measurement of the color intensity was conducted at a wavelength of 450 nm and a reference wavelength between 620 nm and 650 nm and read within 30 min of the stop solution being added. The results were calculated by four-parameter fitting method (Logistic curve fitting four parameters), the standard curve was established with the concentration value of the standard substance on the x-axis, and the logarithm (log) value of the OD value of the standard substance on the y-axis. Substitute the absorbance value into the equation to calculate the corresponding concentration value of serum antibody. The tests were calibrated using WHO international standard serum 06/140, and antibody results obtained from the tests were expressed in international units per milliliter (IU/ml). To avoid confusion, we modify the statement as follows: To avoid test bias, all detection tests were conducted by the same staff members using commercial ELISA kits from Zhengzhou Yit Bio-Tech Co., Ltd., which was previously determined against NIBSC 06/140, and antibody results obtained from the tests were expressed in international units per milliliter (IU/ml). Please see page 6.

  1. The authors should explain in detail how the exact concentration of serum IgG was calculated and provide guidelines for interpreting the results. For example, it is mentioned that <5 IU/ml was considered negative, but what about concentrations between 5-20 IU/ml and >20 IU/ml? Additionally, the rationale for considering ≥80 IU/ml as indicating a recent infection should be provided.

Reply: I am very sorry that I failed to find out and correct this obvious error when uploading the manuscript. According to the manufacturer's instructions,the minimum level of anti-PT IgG detection is 5 IU/ml, anti -PT IgG and anti -FHA IgG antibody concentrations <20 IU/mL, is considered negative. If ≥ 20 IU/mL is judged positive, it is considered to have immune protection to the human body. When the PT-IgG result is ≥ 80 IU/mL, it suggests recent or acute pertussis infection, requiring follow-up with a second specimen after 2-4 weeks. Individuals not vaccinated exhibit elevated serum antibody levels, indicating recent infection. In the first year after vaccination, distinguishing whether antibodies result from self-recognized infection or vaccine induction requires combination with other methods and clinical findings for diagnosis. Please see page 6.

  1. Line 155: It is not clear how the geometric mean concentrations (GMCs) and corresponding seropositivity rates of antibodies were calculated. The authors should explain the methodology in more detail.

Reply: The geometric mean is used to summarize the central tendency of the antibody concentrations within the sample. It is calculated by multiplying all the individual antibody concentrations together and then taking the nth root, where n is the number of individuals in the sample. The formula for calculating the geometric mean is: Here, x1, x2, ..., xn represents the individual antibody concentrations, and n is the total number of individuals in the sample.

seropositivity is defined as having detectable antibody levels above the threshold of 20 IU/mL. So, for each individual's antibody concentration, if it is greater than 20 IU/mL, they are considered seropositive; otherwise, they are seronegative. The seropositivity rate is then calculated as the number of seropositive individuals divided by the total number of individuals in the sample. Please see page 6.

  1. Figure 2: The authors should consider improving the display of the results to make them clearer and easier to understand.

Reply:  Reviewer 1 raised the same issue, we put symbols within the lines to make it easier to interpret as a stand-alone figure.

  1. P8 line 14: The statement about the proportion of individuals with anti-PT IgG antibody levels in different age groups seem not consistent with the results in Table 1. Please carefully review the data.

Reply: In order to make the meaning of the sentence clearer, we modify it as follows: With the exception of the 0-2 months age group, the proportion of individuals with anti-PT IgG antibody levels equal to or greater than 80 IU/mL was highest in the 5-6years age group(5.3%). In all other age groups, this proportion ranged from 2.0% to 3.9%. Please see page 11.

  1. P8 line 17: The authors should specify which group the result of the GMC of anti-FHA IgG antibody and the seropositivity rate corresponds to.

Reply: we modify it as follows: 

The trend of FHA antibody in different age groups was similar to that of PT, but with slight differences. The seropositivity rate and the GMC of anti-PT IgG and anti-FHA IgG antibodies were highest in the 18-23 months age group(31.6% vs 48.7%, 20.33 vs 28.26). In addition, the seropositivity rate of anti-FHA IgG antibodies among individuals who received the full course vaccination (4 doses) was significantly higher compared to incomplete vaccination group(1-3 doses) and non-vaccination group. Please see page 11.

  1. Table 1: The definition of <5IU/ml as negative should be reflected in the appropriate column heading.

Reply: Due to previous negligence, 5 was mistakenly regarded as the cutoff value, which has been modified as follows: According to the manufacturer's instructions, the minimum level of anti-PT IgG detection was 5 IU/ml, anti -PT IgG and anti -FHA IgG antibody concentrations <20 IU/mL, was considered negative. If ≥ 20 IU/mL is judged positive, it is considered to have immune protection to the human body. An anti-PT IgG concentration ≥80 IU/mL was considered to indicate a recent infection if the subject had not received the pertussis containing vaccine within the previous year. Please see page 6.

  1. P8 line 22: “Specifically, the seropositivity rate in the group receiving the full course vaccination was 43.3%, while in other groups it was 24.6% (Table 1)”. 24.6% was for which groups in Table 1?The authors should review the data and revise the statement about the seropositivity rate in different groups to ensure consistency with Table 1. Additionally, they should clearly define how the seropositivity rate was calculated.

 Reply: I am very sorry that this is an obvious mistake that was not caught and corrected in time when the manuscript was uploaded, and we modify it now as follows: 

The trend of FHA antibody in different age groups was similar to that of PT, but with slight differences. The seropositivity rate and the GMC of anti-PT IgG and anti-FHA IgG antibodies were highest in the 18-23 months age group(31.6% vs 48.7%, 20.33 vs 28.26). In addition, the seropositivity rate of anti-FHA IgG antibodies among individuals who received the full course vaccination (4 doses) was significantly higher compared to incomplete vaccination group(1-3 doses) and non-vaccination group. Please see page 11.

  1. P8 line 31: The authors should specify what 17.71 months and 16.95 months refer to which group.

Reply: Because the previous content has introduced the content of Figure 3A and 3B in detail, we only mark after 17.71 months(PT-IgG) and 16.95 months(FHA-IgG).  

The RCS analysis revealed a non-linear relationship between the duration after immunization and the OR of having PT-IgG and FHA-IgG antibody concentrations ≥20 IU/mL in children with documented immunization history (1-4 doses) (Poverall<0. 001, Pnonlinear≤0.001). The inflection points of the RCS curves were at 17.71 months (PT-IgG) and 16.95 months (FHA-IgG) after immunization, respectively, after which the OR of having antibody levels exhibited a gradually increasing and then decreasing non-linear trend (Figure 3A and 3B). Please see page 11.

  1. P8 line 37: Similarly, the authors should provide clarification about the time intervals mentioned (e.g., 7.36 months and 7.56 months) to which group.

Reply: Because the previous content has introduced the content of Figure 3C and 3D in detail, we only mark after  32.54 months (PT-IgG) and 31.29 months (FHA-IgG).

The inflection points of the RCS curves were at 32.54 months (PT-IgG) and 31.29 months (FHA-IgG) after immunization, respectively (Figure 3C and 3D). Please see page 11.

  1. Figure 3: The authors should clearly indicate the unit for the x-axis, which represents the time since the last dose of the vaccine. It would also be helpful to label the subpanels of the figures with keywords to make them easily understandable.

Reply: we modify as follows: 

17.The overall populated tested include the kids with full shot and some with uncompleted shot. The relative low positivity rate of antibody is due to the uncompleted shot or the decline of the antibody? It would be valuable for the authors to conduct further analysis within the group of fully vaccinated children to assess the antibody trends with age. This would help elucidate whether the relative low positivity rate of antibodies is due to incomplete vaccination or a decline in antibody levels.

Reply: The observed relatively low positivity rates of PT-IgG antibodies raise the question of whether they are primarily influenced by the incomplete vaccination status or the natural decline of antibodies over time. According to the data presented in Table 1, the positivity rate for PT-IgG antibodies in children who received the full course of DTaP vaccination is 24.6%, while in those with incomplete vaccination, it is 14.0%. Both of these positivity rates are comparatively low. To understand the reasons behind these lower rates, further analysis is necessary to differentiate the impact of incomplete vaccination from the normal waning of antibody levels over time. By fitting a spline graph of binary logistic regression models using RCS, we found a non-linear association trend between post-immunization duration in children with immunization history and OR values of PT-IgG and FHA-IgG antibody concentrations ≥ 20IU/mL, indicating that the PT-IgG and FHA-IgG antibodies had dropped below protective levels around 17 months after vaccination, while for children who completed the full course of immunization, their antibodies dropped to the lowest level at around 32 months. This finding further suggests that timely and effective full immunization leads to longer antibody duration.

Reviewer 3 Report

Manuscript from Wang and Cols study changes in immunity in children from the province of Jiangsu, China. The measurement of IgG as a marker was used to determine the response and analyzed by the RCS model. We felt that the study was well designed and performed, and the results are interesting since no such kind of report was previously performed. The strongest of the study are reflected in the experimental design and data analysis, which clearly shows the decrement of immune response in the studied group. Also, the discussion on the limitation of results is clear and sound. The only minor thing we felt is in the title. Referring to a study of pandemic time from a long last incidence epidemiological analysis is improper. Please think about another more adjusted.

Author Response

Manuscript from Wang and Cols study changes in immunity in children from the province of Jiangsu, China. The measurement of IgG as a marker was used to determine the response and analyzed by the RCS model. We felt that the study was well designed and performed, and the results are interesting since no such kind of report was previously performed. The strongest of the study are reflected in the experimental design and data analysis, which clearly shows the decrement of immune response in the studied group. Also, the discussion on the limitation of results is clear and sound. The only minor thing we felt is in the title. Referring to a study of pandemic time from a long last incidence epidemiological analysis is improper. Please think about another more adjusted.

Reply: Thank you for your valuable suggestions. This study investigates the epidemiological changes in pertussis in Jiangsu Province from 2007 to 2022. It aims to elucidate the prevalence of pertussis in the region and provide readers with an understanding of the current trends in pertussis occurrence, including the period during the COVID-19 pandemic. The primary focus of this research lies in evaluating pertussis antibody levels in the 0-14 age group by analyzing blood specimens collected from healthy individuals (aged 0-14 years) during the period of 2019-2022 (the COVID-19 pandemic). The data were analyzed using a binary logistic regression model with restricted cubic splines (RCS) to study the dynamic changes in IgG antibody levels after pertussis vaccination. Notably, this study did not compare the impact of the COVID-19 pandemic on pertussis prevalence. Accordingly, the revised title of this manuscript is " The Decay of Pertussis Antibodies in 0-14 Age in Jiangsu, China " please see page 1.